# Alveolar lipids in pulmonary disease. A review

**DOI:** 10.1186/s12944-020-01278-8

**Published:** 2020-06-03

**Authors:** Christina W. Agudelo, Ghassan Samaha, Itsaso Garcia-Arcos

**Affiliations:** Department of Medicine, SUNY Downstate Health Sciences University, Brooklyn, NY 11203 USA

**Keywords:** Lungs, Alveoli, Surfactant, IPF, COPD, Lipids, Type 2 cells, Pulmonary disease, Lipid metabolism

## Abstract

Lung lipid metabolism participates both in infant and adult pulmonary disease. The lung is composed by multiple cell types with specialized functions and coordinately acting to meet specific physiologic requirements. The alveoli are the niche of the most active lipid metabolic cell in the lung, the type 2 cell (T2C). T2C synthesize surfactant lipids that are an absolute requirement for respiration, including dipalmitoylphosphatidylcholine. After its synthesis and secretion into the alveoli, surfactant is recycled by the T2C or degraded by the alveolar macrophages (AM). Surfactant biosynthesis and recycling is tightly regulated, and dysregulation of this pathway occurs in many pulmonary disease processes. Alveolar lipids can participate in the development of pulmonary disease from their extracellular location in the lumen of the alveoli, and from their intracellular location in T2C or AM. External insults like smoke and pollution can disturb surfactant homeostasis and result in either surfactant insufficiency or accumulation. But disruption of surfactant homeostasis is also observed in many chronic adult diseases, including chronic obstructive pulmonary disease (COPD), and others. Sustained damage to the T2C is one of the postulated causes of idiopathic pulmonary fibrosis (IPF), and surfactant homeostasis is disrupted during fibrotic conditions. Similarly, surfactant homeostasis is impacted during acute respiratory distress syndrome (ARDS) and infections. Bioactive lipids like eicosanoids and sphingolipids also participate in chronic lung disease and in respiratory infections. We review the most recent knowledge on alveolar lipids and their essential metabolic and signaling functions during homeostasis and during some of the most commonly observed pulmonary diseases.

## Introduction

The lung is seldom considered a lipid metabolic organ. However, it does sustain active lipid metabolism, especially in the alveolar area, where surfactant homeostasis is exquisitely regulated to ensure continuous optimal function in each respiration cycle. Surfactant is a lipoprotein complex, composed mostly of phospholipid, and it is an absolute requirement for gas exchange. It has been known for a long time that the cause of infant respiratory distress syndrome (IRDS) and death of premature infants is surfactant insufficiency, and exogenous therapy is nowadays a standard of care in these cases (Table 1). Both animal derived and synthetic surfactants provide clinical benefits and decrease mortality of preterm infants with IRDS. For expanding on the lipid pathology of IRDS, the reader is referred to the existing revisions in the literature ([1] and references herein).

**Table 1:** Overview of lipid changes in common pulmonary conditions

**Pulmonary Condition**	**Lipid Process**	**Reference**
Infant Respiratory Distress Syndrome (IRDS)	Surfactant Insufficiency	[1, 65, 74, 208, 225, 227, 232, 233, 235, 239, 240, 244, 245]
Acute Respiratory Distress Syndrome (ARDS/RDS)	Neutral Lipid Accumulation	[51]
	Surfactant Lipid Deficiency	[27, 67–69, 247, 248]
	Increased PL-Mediated Fibrin Polymerization	[247]
	Protective Role of Sphingolipid Signaling	[249–251]
Acute Lung Injury (ALI)	T2C Damage	[134, 252, 253]
	Surfactant Lipid Alterations	[134, 242, 252, 253]
	Dysregulated Lipid Transport	[177, 178]
	Protective Role of Sphingolipid Signaling	[249–251]
Chronic Obstructive Pulmonary Disease (COPD)	Surfactant Lipid Deficiency	[6, 7, 67, 88–91, 95, 98–104]
	Disrupted Reverse Lipid Transport	[105, 106]
	T2C Damage	[92–98, 103, 107, 108]
	Disrupted Alveolar Architecture	[104]
	Impaired AM Sphingolipid Signaling	[110–113]
Vaping-Associated Lung Injury	Intracellular and Luminal Lipid Accumulation	[115–120, 194, 195]
	Dysregulated AM Lipid Metabolism	[196]
Idiopathic Pulmonary Fibrosis (IPF)	Surfactant Lipid Alterations	[67, 129–134, 139–141, 143–145, 148]
	Downregulated T2C Lipid Metabolism	[125–128, 139–141, 148, 150]
	T2C ER Stress	[147–149, 151]
	T2C Damage	[135–137]
	Dysregulated AM Lipid Metabolism	[130, 138, 142]
	Dysregulated Eicosanoid Production	[148, 152–156, 158–163, 165–168]
	Dysregulated Sphingolipid Signaling	[169–174]
	Decreased Alveolar Surface Area	[129]
Pulmonary Alveolar Proteinosis (PAP)	Luminal Surfactant Accumulation	[6, 63]
	AM Cholesterol Accumulation	[59–64, 191]
Pneumonia	Surfactant Lipid Alterations	[67, 175]
	Dysregulated Lipid Transport	[176]
	Host-Pathogen Lipid Interaction	[179–190]
	Alveolar Cellular Damage	[193]
Influenza	Lipid-Mediated Host Defense	[5]
	Host-Pathogen Lipid Interaction	[205, 206]
Tuberculosis (TB)	Host-Pathogen Lipid Interaction	[210, 211, 215–218]
	Host Eicosanoids Differentially Affect Pathogenesis	[212–214]
SARS and SARS-CoV-2	Diffuse Alveolar Damage	[222, 223]
	T2C Hyperplasia	[221, 222]

Beyond their essential roles as surfactant, energy storage and structural components, different lipids can also exert different signaling functions during physiological and pathophysiological processes. In the lung, lipids were intensely studied in the context of surfactant metabolism during the second half of the twentieth century and are now garnering new interest in multiple disease contexts, partly owing to the latest development of sophisticated and sensitive methods for detection and data analysis. Currently ongoing research on pulmonary fibrosis and electronic cigarette-induced lung injury highlights the relevance of pulmonary lipids during disease.

In this review, we focus on adult pulmonary disease to give a consolidated view of the most updated literature on alveolar lipids. Reviewing the roles of all lipid species in all pulmonary cell types in every form of disease would be excessively lengthy and exceed our scope. Instead, this paper is focused on the alveolar area, which is the most studied and where the major pulmonary lipid metabolic cells reside. However, we are convinced that lipid metabolism will reveal itself of interest in any other lung region and cell type considered.

## Alveolar cellular environment and surfactant biology

The alveolar epithelium is composed of alveolar type 1 (T1C) and type 2 cells (T2C). Type 1 cells cover most of the alveolar surface and are highly specialized in performing the gas exchange between blood and air. T1C also participate in interactions with alveolar macrophages, and modulation of fibrotic responses [2, 3]. At the junctions of the alveolar sacs reside the T2C, whose main function is the production of surfactant.

Pulmonary surfactant is probably the best-known lipid complex in the lung, and it is an absolute requirement for respiration. Surfactant reduces surface tension during inspiration and prevents alveolar collapse at the end of expiration. Alveolar T2C are the major lipid metabolic cells of the lung because they need to orchestrate a complex set of lipid metabolic pathways to effectively adjust surfactant synthesis, secretion and recycling in different physiologic situations. Pulmonary surfactant is a lipoprotein complex, with 90% of its mass being lipid and the remaining 10% proteins that are in many cases specific of the alveolar compartment. Amongst the 90% lipid, the large majority is phospholipid (PL), especially phosphatidylcholine (PC) and more specifically dipalmitoyl phosphatidylcholine (DPPC), the main lipid species responsible for the surface tension-reduction properties of the surfactant mixture. Phosphatidylglycerol is capable of modulating macrophage function and it is used as a marker of lung maturity [4]. Palmitoyl-oleoyl-phosphatidylglycerol and phosphatidylinositol (PI) can antagonize Toll-like receptor (TLR) activation. TLR activation is a crucial step in the virulence of certain viruses such as Influenza A and Respiratory Syncytial Virus [5], underscoring the role of phosphatidylglycerol and PI in controlling viral infections and the associated inflammatory cascades [5]. Other lipids of smaller abundance in alveolar surfactant include cholesterol, sphingolipids and plasmalogen phospholipids [6, 7].

**Fig. 1:**
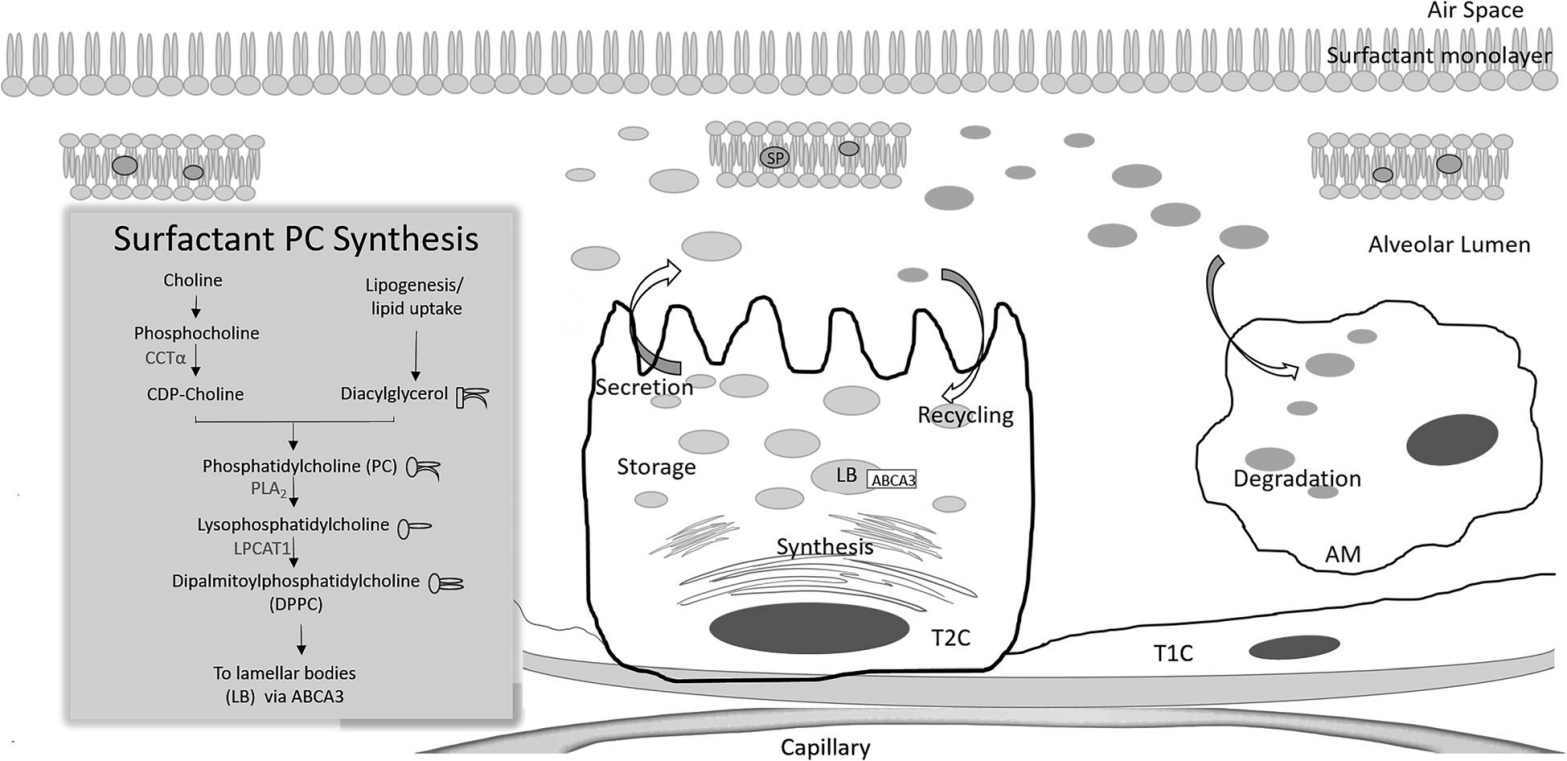
Surfactant lipid synthesis and exocytosis. Simplified scheme of intracellular pathways leading to de novo synthesis of DPPC and its routing to lamellar bodies, from where it will be released into the alveolar lumen, where it will be used, recycled and degraded. For the synthesis, CDP-choline and diacylglycerol are coupled to form PC. A large proportion of PC is remodeled to render DPPC. Surfactant is stored in lamellar bodies until secretion to the alveolar lumen, where it organizes in bilayers and monolayers (see text for further detail). The surfactant life cycle is completed by its recycling by T2C or degradation by AM. T1C: type 1 cell; T2C: type 2 cell, AM: alveolar macrophage; LB: lamellar body; SP: surfactant protein; PC: phosphatidylcholine; DPPC: dipalmitoylphosphatidylcholine; CCTα: CTP:phosphocholine cytidylyltransferase alpha; PLA_2_: phospholipase A2; LPCAT1: lysophosphatidylcholine acyltransferase 1.

Surfactant lipid metabolic genes are transcriptionally regulated by the same factors governing lipid metabolism and lipogenesis in other tissues [8], and in T2C, surfactant lipid synthesis and secretion are coupled with lipid availability [9]. T2C obtain lipid precursors from the plasma using proteins capable of transporting and interacting with lipids, such as CD36 and glycosylphosphatidylinositol-anchored high density lipoprotein–binding protein 1 (GPIHBP1) [9–16]. De novo lipogenesis can also contribute to the intracellular pool of fatty acids (FA) in T2C. As in other eukaryotic cells, the synthesis of PC in T2C occurs mainly through the Kennedy pathway (Fig. 1). The rate limiting enzyme of this pathway is CTP:phosphocholine cytidyltransferase alpha (CCTα). Newly synthesized PC usually contains a monounsaturated FA in position *sn*-2, and incorporated PC can have heterogeneous acyl composition. Hence, PC needs to undergo remodeling by phospholipase A_2_ (PLA_2_) and lysophosphatidylcholine acyltransferase 1 (LPCAT1) through the Lands cycle to render DPPC. Intracellular DPPC is then transported into a specialized organelle, the lamellar body, through the specific transporter ABCA3 and stored there until secretion to the alveoli [17–19]. Mouse models with loss of function for ATP-binding cassette A1, G1, or A3 (ABCA1, ABCG1, ABCA3) or CCTα all show surfactant insufficiency, pulmonary intracellular accumulation of lipid, and inflammation [20–26]. In humans, impairment of lipid metabolism in pulmonary T2C causes surfactant insufficiency resulting in deficient pulmonary function. For example, ABCA3 loss of function results in neonatal respiratory distress syndrome and defective lamellar body synthesis [27].

In the alveolar lumen, surfactant reduces the surface tension in the alveolar walls from 70 mN/m to nearly 1mN/m [28]. The biophysical properties and extracellular metabolism of secreted surfactant are complex and the exact mechanisms for surfactant ability to reduce surface tension during alveolar expansion and to support high levels of pressure during lateral compression at the end of expiration are still undergoing intense research [29, 30]. Surfactant secretion by T2C occurs by exocytosis, with lamellar bodies fusing their limiting membrane with the apical plasma membrane and releasing their content into the alveolar lumen (reviewed in [31]). The content of the lamellar bodies is initially detected in the alveolar spaces as densely packed lamellar body-like particles that are thought to organize in multiple phospholipid bilayers and monolayers when they reach the alveolar surface [32]. In vitro and ex vivo experiments with clinically used surfactant and with simplified model phospholipid mixtures have shown the coexistence of two domains in the pulmonary surfactant film at physiological temperatures: bilayers enriched in unsaturated phospholipids and monolayers mainly composed of DPPC. A “squeeze-out” mechanism has been proposed. Compression of the film during expiration would result in the formation of multilayers of phospholipids that would re-spread during inspiration. These reversible transitions between bilayer and monolayer formations seem to be enabled by the hydrophobic surfactant proteins B and C (SP-B and SP-C) [33–35]. The interactions between lipids and proteins are complex and have been discussed in detail elsewhere [36].

Surfactant-specific proteins account for ~ 10% of the lipoprotein complex by mass, but they affect the behavior of the surfactant lipids. The most abundant and studied proteins are Surfactant Proteins A, B, C and D (SP-A, SP-B, SP-C and SP-D). SP-A and SP-D are hydrophilic and participate in the immune function roles of surfactant, as they can bind exogenous pathogens and facilitate their clearance by alveolar macrophages. Indeed, SP-A and SP-D knockout mice are more susceptible to infections and other lung diseases [37–40]. In contrast, SP-B and SP-C are hydrophobic and participate in surfactant lipid film dynamics. Once it is secreted into the alveolar lumen with the rest of the components of the lamellar bodies, SP-B is adsorbed in the air liquid interface. Extracellularly, SP-B participates in stabilizing the lipid film at all stages of the respiratory cycle [41]. SP-C is an exclusive constituent of pulmonary surfactant. SP-C also acts at the lipid film and participates in enabling the lipid to be compressed in a manner compatible with subsequent re-spreading during inspiration [42, 43]. SP-C knockout models develop profound alterations in surfactant lipid metabolism, discussed in the following pages in the context of disease [44–46].

Cholesterol comprises 5–10% of surfactant lipid. Experiments using native surfactant material showed that cholesterol was required for effective spreading properties of the lipid monolayer, as well as maintenance of the lateral structure, with differentiated lipid domains [47]. It was initially thought that cholesterol impaired the surface tension activity of surfactant, leading to its exclusion in the initial preparations clinically used for premature infants. However, subsequent experiments showed that cholesterol inhibits film compression and surface tension activity only at 20% and higher concentrations in the surfactant [48]. Elevated levels of cholesterol in the surfactant mixture has been proposed to be a mechanism for ventilator-induced lung injury [49] and cholesterol crystals have been found in the bronchoalveolar lavage (BAL) of idiopathic pulmonary fibrosis (IPF) patients [50]. Patients with acute respiratory distress syndrome (ARDS) also showed increased neutral lipid amounts in their BAL and this elevation persisted after exogenous surfactant administration. In vitro, neutral lipid supplementation of a clinically used or natural surfactant reduced surface tension properties, with monoglycerides and cholesterol exhibiting the greatest inhibitory effects [51]. Multiple experiments have consistently shown the ability of neutral lipids to disrupt surfactant function [48, 49, 52, 53]. Neutral lipids may function as barriers to surfactant therapy efficacy, hence further contributing to pathology. In a clinical study, the therapeutic benefits of exogenous surfactant administration in ARDS were hampered by accumulation of cholesterol in the alveolar space [51].

Extracellular surfactant can be degraded by alveolar macrophages (AM) or recycled by T2C during physiological conditions (Fig. 1) [17]. AM make up 90 to 95% of the cells collected by BAL [54], and they degrade surfactant lipids [55]. This function can be essential also during pathological conditions when the lung is exposed to oxidizing agents that can induce oxidation of surfactant proteins and lipid, as well as lipid aggregation [56, 57]. Granulocyte macrophage colony stimulating factor (GM-CSF) is required for macrophages’ ability to clear surfactant, and deficiency in GM-CSF can lead to excessive surfactant accumulation and pulmonary alveolar proteinosis (PAP) [58]. Indeed, PAP is characterized by abnormal accumulation of surfactant in the alveoli and the terminal airways. Quantitative lipidomics of BAL of patients with PAP showed a significant increase in both free cholesterol and cholesterol esters. Other surfactant components, including sphingolipids, ceramides, PE, PS, PC, LPC and the surfactant proteins were also increased [6]. The clinical course is heterogeneous, ranging from spontaneous resolution to death due to respiratory failure [59]. In the absence of GM-CSF signaling, AM massively accumulate intracellular cholesterol-rich lipid droplets and extracellular surfactant also showed an increased proportions of cholesterol. This points out at defects in GM-CSF signaling and AM cholesterol clearance as the primary drivers of PAP [60–62].

The most effective and proven treatment for PAP is whole lung lavage [63], and GM–CSF can be used as inhalation therapy, or subcutaneous injection if whole lung lavage fails or is contraindicated. Experimentally, inhibition of cholesterol synthesis by statins showed positive therapeutic value. Since the main driver of PAP is defective cholesterol clearance by AM and not increased synthesis, the mechanism for the observed effects is not clear [64].

On the other end of the spectrum is surfactant deficiency, the cause of neonatal IRDS [65, 66]. But deficiency and alterations in surfactant lipids and proteins have long been described also in adult pulmonary diseases, including IPF, adult onset PAP, ARDS and lung cancer [27, 67–69]. Genetic alterations in components of the surfactant metabolism pathway have detrimental effects on pulmonary function and disease pathogenesis. Mutations in the genes encoding SP–A, *SFTPA1* and *SFTPA2*, are associated with interstitial lung disease and increased susceptibility to adenocarcinoma of the lung [70, 71]. Autosomal recessive mutations in the gene encoding SP–B, *SFTPB*, were among the earliest described [72, 73]. SP–B–deficient humans and mice develop respiratory distress and respiratory failure independent of surfactant lipid composition [74–76]. SP–B knockout mice show aberrant lamellar body formation, and incorrect SP–C intracellular processing [75]. The lack of SP–B disturbs lamellar body formation in T2C, impairs processing of other surfactant proteins, and disrupts surfactant recycling [77]. Deficiency of ABCA3 also recapitulates this phenotype and causes respiratory failure [78, 79]. Consistently, mutations in the homeodomain–containing transcription factor TTF–1, master regulator of surfactant protein and *ABCA3* expression, were also associated with respiratory failure [80]. Finally, SP–C mutations were also associated with increased susceptibility to pneumonitis and emphysema due to decreased SP–C–mediated alveolar surfactant spreading [45]. Other genetic abnormalities or deficiencies in SP–C lead to cholesterol accumulation in the alveoli and are described in detail in the following pages [46, 81].

Although we focus on the lipid aspects of alveolar cell function in this review, it is worth mentioning that alveolar cells partake in other processes. T2C contribute to other essential roles for pulmonary homeostasis and alveolar repair [82], as well as in immune defense processes [83] and inflammation [84, 85]. Interstitial macrophages are also crucial in regulating and suppressing unwanted or excessive immune reactions [86]. During pathogenic processes, fibroblasts recruit inflammatory cells, and partake in remodeling and regenerating the extracellular matrix after tissue injury. Excessive activation of fibroblasts can lead to fibrosis and scarring of the lung [87]. The role of lipid mediators in the development of fibrosis and other adult and chronic pulmonary diseases is discussed in the following pages.

## COPD and other smoking–related diseases

The pathophysiology of chronic obstructive pulmonary disease (COPD), which involves emphysematous destruction of alveolar sacs and airway remodeling, is highly dependent on environmental and genetic factors. Cigarette smoking is among the most prevalent pulmonary public health concerns worldwide and is a leading cause of COPD in smokers and former smokers, although other causes, including exposure to environmental pollutants, also contribute significantly to this burden.

COPD patients have both decreased surfactant amount and altered surfactant composition, potentially making it less effective at reducing the surface tension (Table 1) [6, 7, 88]. Our group recently reported the decrease in total surfactant lipid, as well as specific lipid species, in patients with COPD [7]. Decreases in total BAL lipid, total PL, PC 30:0, PC 32:0, and total cholesterol, amongst other lipids, strongly correlated with decreased lung function. The main surfactant lipid changes observed in COPD patients were replicated in a mouse model exposed to 6 months of second–hand smoke, which enables future mechanistic research. This study is well aligned with prior ones showing that smoking reduced BAL PL content in humans [89]. Surfactant replacement therapy provided pulmonary function improvement in a small clinical trial in individuals with stable bronchitis, often a component of COPD [90]. However, the mechanism for this improvement, as well as the roles of surfactant lipids in COPD, are not clear [67, 91].

COPD and emphysema can result in decreased alveolar T2C or premature senescence, potentially impacting lipid metabolism (Table 1) [92, 93]. Cigarette smoking can affect lung lipid homeostasis through direct and indirect mechanisms. The mechanisms for smoke exposure–induced damage to the T2C include inflammation, oxidative stress, dysfunctional DNA repair mechanisms, and proteolysis–antiproteolysis imbalance, amongst others [94–96]. Indeed, T2C of individuals with emphysema have increased reactive oxygen species generation and DNA damage when compared to those of healthy controls [97]. In T2C–derived human A549 cells, cigarette smoke induced apoptosis, inhibited proliferation, and spurred epithelial–mesenchymal transition (EMT) [98].

Smoke exposure damaged T2C and caused alterations of surfactant secretion and composition in multiple animal models [95, 99–104]. Most studies report decreased surfactant lipid availability after chronic exposures to smoke, a common model of COPD. Rats exposed to 60 weeks of nose–only smoke showed significant decreases in BAL DPPC content when compared to room air controls [100]. BAL from smoke–exposed rats had excessive surfactant compressibility and reduced re–spreadability. A mechanism for smoke decreasing surfactant levels can be direct damage to the T2C, including alterations in cell adhesion, proliferation and direct cell lysis.

At the molecular level, there are many potential mechanisms of smoke–mediated disruption of surfactant metabolism. Smoke was found to directly interfere with DPPC synthesis by reducing PLA_2_ activity by more than 50% [101], and both in humans and mice, smoking caused alterations in pulmonary expression of proteins implicated in reverse lipid transport, including ABCA1, ABCG1, ApoE and SRB1 [105, 106]. Cultured T2C acutely exposed to cigarette smoke extract showed inhibition of stimulated PC secretion, while basal PC secretion was not affected [99]. Another mechanism by which cigarette smoke may interrupt surfactant metabolism is by the generation of free radicals, and enhancing oxidative stress, which in turn can elevate the levels of TNFα, and other proinflammatory markers [107, 108] [103]. In addition, nicotine can induce excessive expression of SP–A and SP–C in an embryonic mouse lung culture [109]. In addition to T2C damage, cigarette smoke exposure also affect the whole lung parenchyma. In addition to alveolar congestion following smoke exposure, one study reports diffuse alveolar collapse, septal hypertrophy, and other gross lung abnormalities [104].

AM are also affected by cigarette smoke (Table 1). Tran et al. [110] showed that AM exposed to cigarette smoke had less efficient efferocytosis, and this was attributed to impaired sphingosine kinase (SPK) activity. Similarly, in vivo instillation of ceramide impaired AM efferocytosis, and this effect was reproducible by sphingosine treatment, suggesting the involvement of ceramidase activity in this impairment [111]. Efferocytosis is necessary for elimination of airway apoptotic cells that if not removed, can eventually necrotize and lead to chronic inflammation [112]. Both cigarette smoking and electronic cigarettes disrupted the endothelial barrier by affecting intracellular ceramides, mitogen activated protein kinase (MAPK) activation and myosin light chain phosphorylation [113].

The use of electronic cigarettes and other “vaping” devices has become popular in recent years. Electronic cigarette use can have deleterious effects on lung lipid metabolism regardless of nicotine content (Table 1). The use in mice of an electronic nicotine delivery system (ENDS) during 4 months lead to increased deposition of surfactant in the alveoli without increase in inflammatory markers or emphysema, and interestingly, there were no differences between the nicotine–containing and vehicle–only groups when compared to air–exposed mice. Exposure to ENDS increased cytoplasmic lipid droplets in AM and increased the number of lamellar bodies with disrupted architecture in T2C. In addition, the expression of ABCA1 and ABCG1 was significantly decreased, together with the expression of surfactant proteins SP–A and SP–D. When challenged with influenza infection, ENDS–treated mice showed disrupted innate immunity and enhanced inflammation, with persistent expression of interferon–γ (IFN–γ) and tumor necrosis factor–α (TNF–α) [114].

In November 2019, a public health concern was raised following the report of multiple cases of e–cigarette and vaping associated lung injury requiring hospitalization with some cases being fatal, and with high incidence amongst teenagers and young adults. The symptoms included dyspnea and hypoxemia with no associated infection, and in multiple cases the BAL showed increased accumulation of neutral lipids in AM. While the mechanisms for the lung injury have not been deciphered at the moment of writing this article, multiple cases seem to be associated with vaping of cannabinoid and other terpene–rich oils that may have been customized by the final users [115–120]. In a histopathologic study of lung biopsies obtained from vaping–induced lung injury cases, acute lung injury was confirmed and foamy AM and pneumocyte vacuolization were observed in all the samples [118]. The potential effects of lipid–vapor inhalation in alveolar lipid metabolism are still largely unknown.

Other particulate and gaseous environmental agents have also been linked to altered surfactant lipids. Chronic inhalation of silica dust causes silicosis, which is characterized by foamy macrophages. These AM have higher intracellular levels of neutral lipids and show elevated mRNA levels of the lipid receptor CD36 and transcription factor PPARγ [121]. Inhaled ozone (O_3_) generated from nitrogen oxides and volatile hydrocarbons alter lung function and cause inflammation [122]. Once inhaled, approximately 80% of O_3_ remains in the lungs and may react with the carbon–carbon double bonds of surfactant phospholipids and cholesterol, a process termed “lipid ozonation” [122]. In addition, common household dust mites have also been linked to the generation of proinflammatory eicosanoids and cytokines in alveolar macrophages [123].

## Lipids in interstitial lung disease and idiopathic pulmonary fibrosis

IPF is a rare disease that has attracted attention lately due to the approval of the first few drugs for its treatment. IPF patients experience a progressive decline in pulmonary forced vital capacity (FVC), with their lung parenchyma and airways showing extensive remodeling, fibroblast proliferation, excessive extracellular matrix deposition and loss of compliance. Together, the clinical manifestation is shortness of breath and difficulty in lung inflation. The progression after diagnosis is fast, with death occurring in 3–10 years unless a transplant is provided. The currently approved drugs decelerate the disease progression, but there is no regression.

A main difficulty in IPF treatment is the lack of markers for early disease detection. In an attempt to identify a biomarker, a small study showed increased LysoPC in the serum of IPF patients, and this provided a rationale for the development of autotaxin inhibitor drugs [124]. However, there are no specific pulmonary biomarkers. Another difficulty in IPF treatment lies on the current lack of knowledge on early triggers and clear mechanisms for disease progression before the end–stage, when patients normally present extensive remodeling.

Recent studies using single cell RNA sequencing have shed some light on the pathobiology of IPF. Reyfman et al. [125] detected over 500 genes differentially expressed in patients with IPF. Interestingly, in T2C the top 500 genes downregulated in IPF were all included in lipid metabolic pathways, including “sterol/steroid biosynthetic process”, “cholesterol metabolic process” and “lipid metabolism process”. AMs also showed profound alterations in lipid metabolism, with downregulated pathways including “cellular response to fatty acids” and “positive regulation of lipid metabolic processes”. It is not clear if this loss of ability to handle lipid metabolism by alveolar cells constitutes a cause or a consequence of the disease. The data were confirmed with further qPCR analysis and by other studies [126–128], and these pathways actually comprised the IPF RNA signature of the whole lung when different cell types were not grouped separately.

Naturally occurring mutations can confer susceptibility to fibrosis development later in life. Mutations in ABCA3, like the most common missense mutation E292V, result in IPF development. Mice carrying this mutation showed chronic surfactant insufficiency, with decreased PL in BAL. T2C showed increased number of lamellar bodies, but of smaller volume, and decreased alveolar surface area. BAL cells, mostly AMs, were increased and there were numerous tissue morphological changes, including peribronchial immune infiltrates and a combination of fibrotic and emphysematous regions. Bleomycin instillation, a common model of experimental fibrosis, generated worse fibrosis and higher morbidity in mutant than in WT mice [129]. Mutations in surfactant protein C (SP–C) also result in IPF development. Nureki et al. [81] generated tamoxifen–inducible knock–in mice expressing the substitution of isoleucine by threonine at codon 73, a mutation associated with familial IPF (SP–C^I73T^ mice). SP–C^I73T^ mice had overexpression of SP–C and developed premature fibrosis that recapitulated the human phenotype, with collagen deposition, T2C hyperplasia, fibroblast proliferation, and decreased compliance. Intracellular lipid metabolism and surfactant lipids were not assessed in this model, and it remains unknown so far if the ability of T2C from SP–C^I73T^ mice to sustain normal lipid homeostasis is affected. In addition to the findings above, SP–C was recently reported to modulate alveolar lipid homeostasis during development of fibrosis. SP–C knockout mice, aside from to developing spontaneous lung fibrosis, also showed AM cholesterol accumulation. In vitro, addition of SP–C to cholesterol–containing vesicles in an AM cell line increased expression of genes involved in cholesterol metabolism and transport [46]. These are the first models of spontaneous lung fibrosis and are valuable tools for the study of IPF pathogenesis.

In agreement with the observations in human IPF, bleomycin–induced fibrosis caused decrease in mRNA of lipogenic genes in whole lung and specifically in T2C [130]. Intracellular lipids including cholesterol, free FA, triglycerides and PL were decreased, as well as ABCA3 mRNA expression. However, BAL lipids were increased. Consistently with a decreased intracellular lipogenic program, T2C showed increased phosphorylation of AMP kinase and acetyl–CoA carboxylase, and they were more glycolytic, with decreased intracellular ATP levels and increased lactic acid production. In vitro experiments with T2C showed that bleomycin induced lipid secretion and increased extracellular ATP, a known surfactant secretagogue, while surfactant lipid reuptake was impaired. Treatment of cultured AM with lipid extracts from BAL of bleomycin–treated mice resulted in foam cell formation. Treatment with oxidized PC was by itself capable of increasing mRNA expression of transforming growth factor beta (TGFβ) and M2 markers in cultured macrophages. These observations led the authors to propose a model where the increase in TGFβ1 and collagen deposition secondary to bleomycin injections causes accumulation of abnormal lipid surfactant in the alveolar space. Accumulated PC can then become oxidized and induce AM transformation into foam cells, further contributing to the profibrotic phenotype generating a feedforward loop. The extent of the overlap of this mechanism with the observed lipid metabolic RNA signature of human IPF patients remains to be determined, but in any case, this work shows a dysregulation of lipid homeostasis that integrates three different alveolar compartments, T2C, alveolar space and alveolar macrophages, during the pathogenesis of pulmonary fibrosis.

Experimental bleomycin exposure results in alterations in alveolar lipids, but there is no consensus on the specific changes and directions in different models of experimental fibrosis. Similarly, human studies have reported seemingly contradictory data regarding the direction and magnitude of change in surfactant lipids during disease, and there is no agreement on a mechanism for the observed alterations [67] (Table 1). In some studies, increased levels of PC, cholesterol and bis monoacyl phosphoglycerate (BMP) in whole lung tissue and of all lipids in BAL up to 21 days after bleomycin have been reported [131]. Intratracheally administered bleomycin increased cholesterol and free FA in BAL in rats, and this was associated with increased collagen deposition and epithelial cell proliferation, elastic recoil and surface tension of the BAL. In these studies, the acyl composition of the cholesterol esters in BAL was prominently 16:0, 18:2, 18:1 and 18:0 and it did not change with bleomycin treatment [132, 133].

Pulmonary fibrosis induced by administration of the antiarrhythmic compound amiodarone caused hyperplasic T2C that accumulated PL, BMP and surfactant proteins, and this was associated with ER stress and enhanced pro–apoptotic response. In contrast to bleomycin treatments, amiodarone–induced fibrosis decreased extracellular surfactant DPPC and proportionally increased unsaturated species of PC. Similarly, phosphatidylglycerol decreased and plasmalogen phosphatidylethanolamine increased in BAL [134].

DNA damage and telomere shortening in T2C induced pulmonary fibrosis and decrease survival in mice [135] (Table 1). T2C genetic deletion of telomeric repeat factor 1 (TRF1), a protector of telomere ends, induced pulmonary fibrosis by increasing cellular senescence and apoptosis. In addition, bleomycin treatment in the setting of telomerase–deficiency also recapitulated this fibrotic phenotype [135]. Mice overexpressing telomerase in T2C showed increased T2C proliferation and downregulation of fibrotic and inflammatory gene expression [136]. Aberrant telomere shortening has previously been associated with alveolar stem cell dysfunction [137], and these new data show how telomere dysfunction and subsequent T2C–depletion could be used as a model of IPF.

Bleomycin, silica and radiation exposures all showed deranged lipid metabolism in AMs [130] (Table 1). They accumulated neutral lipid as well as phospholipid, and showed increased mRNA expression of lipid transporters CD36, scavenger receptor A (SRA), ABCA1, ABCG1 and its upstream regulator LXRα. There was an increase in oxidized PC both in BAL and intracellularly in the alveolar macrophages. Simultaneously with these events (14 days after bleomycin treatment), terminal airspaces started to show macrophage infiltration, and progressively increased mRNA expression of TGFβ1 and collagen 1a1. Chronologically, histochemical and biological onset of fibrosis occurred after AM lipid accumulation had started. Fibrosis subsequent to nitrogen mustard exposure triggered the transformation of AM into foam cells [138], and in this case, lipid–laden pulmonary macrophages also showed altered lipid handling pathways as analyzed by RNAseq.

Despite the uncertain direction of the BAL lipid changes during IPF, administration of extracellular surfactant lipid was attempted to improve pulmonary compliance. In mice with bleomycin–induced fibrosis, surfactant replacement therapy rescued compliance and inspiratory capacity, and the number of open alveoli was strongly correlated with static compliance [139]. Overexpression of TGFβ1, which occurs naturally during IPF progression, was associated with loss of apical membrane in T2C during experimental fibrosis [140]. Pretreatment with commercially available surfactant (Curosurf) improved lung mechanics and tissue elastance, increased the number of open alveoli, and preserved the apical membrane surface in T2C. This raises the question of what is the mechanism of alveolar surfactant to protect against the deleterious effects of TGFβ1 treatment. Decreased BAL surfactant proteins, as well as their intracellular mRNA and that of the lipid transporter ABCA3 preceded morphological remodeling of TGFβ1–treated mice, pointing at the role of intracellular T2C lipid metabolism regulation in the development of fibrosis [141]. Through a different mechanism, DPPC and Survanta for 24 and 48 h increased eicosanoid synthesis and inhibited thromboxane A2 synthesis in silica dust–treated AMs [142].

Intracellular lipid mismanagement also partakes in fibrosis development (Table 1). IPF patients and mice treated with bleomycin show decreased expression and activity of multiple lipid metabolic enzymes. Elongation of very long chain fatty acids protein 6 (Elovl6) is one of them. Elovl6 catalyzes the elongation of C16 fatty acids to longer acyl chains and renders unsaturated fatty acyl chains. Elovl6^−/−^ mice are protected from HFD–induced hepatic steatosis and fibrosis, potentially due to their higher palmitoleic/palmitic acid ratio. Alveolar T2C also express Elovl6, and when the Elovl6^−/−^ mice were treated with bleomycin, they developed worse fibrosis, with more collagen deposition and increased mortality [143]. The pulmonary FA composition was altered in Elovl6^−/−^ mice, with a higher proportion of palmitic acid C16:0, in detriment of palmitoleic C16:1(n–9), and this effect was more pronounced after bleomycin. Treatment of cultures of a T2C cell line with palmitic acid triggered apoptosis and increased TGFβ1 expression, both of which were attenuated by treatment with unsaturated fatty acids oleic or linoleic acid. The authors proposed that the increase in palmitic acid content resulted in profibrotic events such as increased TGFβ and apoptosis through increased intracellular generation of reactive oxygen species. Increased FA content, including palmitic acid has been encountered in IPF lungs [144, 145], and treatment of cell cultures with palmitic acid triggered ER stress and apoptotic responses. In vivo, mice pretreated with 2–weeks of high fat diet (HFD) had higher collagen content upon bleomycin treatment. However, it is difficult to assess the contribution of increased extracellular availability of lipid and subsequent pulmonary lipid metabolism to this phenotype, since HFD is known to trigger multiple systemic responses, including low–grade inflammation prior to the onset of obesity [146].

ER stress can serve as a priming event to pulmonary fibrosis by affecting intracellular lipid events. For example, enhanced autophagy in Golgin A2 (GOLGA2)^−/−^ mice limited the subcellular availability of functional mitochondria and lamellar bodies, and this was associated with decreased DPPC and a mild increase in extracellular matrix (ECM) deposition in both lungs and liver [147]. Intranasal tunicamycin increased ER stress, as well as expression of lipogenic enzymes fatty acid synthase (FAS), stearoyl–CoA desaturase 1 (SCD1) and diglyceride acyltransferase (DGAT), their upstream regulator SREBP1, and intracellular triglyceride and PL content [148]. Loss of mitochondrial mitofusin 1 or 2, as well as inhibition of FAS in T2C, worsened bleomycin–induced fibrosis and was associated with perturbed surfactant lipid metabolism [149]. Silica treatment also resulted in a similar lipid synthetic signature that could be rescued by treatment with LXR agonist TO901317, which has been shown to be antifibrotic in other studies [150]. Dysregulated intracellular lipid metabolism can be a cause and a consequence of ER stress. The sole inhibition of SCD1 was enough to induce ER stress and collagen deposition. It was not determined if the potentially increased proportion of saturated fatty acids, including palmitic, resulting of SCD1 inhibition could contribute to this ER stress [148, 151]. Lipid metabolic pathways are often challenging to interpret unless direct metabolite measurements are performed, since same fatty acid substrates can result in different products, with different implications in metabolic and inflammatory pathways, highlighting the relevance and complexity of metabolic fluxes in different cellular conditions.

Activation of PLA_2_ and its action on membrane PC can release arachidonic acid (AA), which serves as a precursor for eicosanoids, potent signaling lipids. AA can be further processed by three different pathways. The cyclooxygenase pathway leads to the generation of prostaglandin H and its derived prostaglandins and thromboxanes, collectively called prostanoids. AA metabolism by the lipoxygenase (12/15 LOX) pathway generates leukotrienes and lipoxins, amongst other lipids, and metabolism by the epoxygenase P–450 pathway generates epoxyeicosatetraenoic acids. The functions of eicosanoids in IPF have been previously summarized in the literature [152, 153] and here we will only briefly mention the latest updates.

Prostaglandins (PGs) and other cyclooxygenase 2 (COX2)–derived prostanoids seem to be protective against experimental fibrosis. In mice, COX2 but not COX1 deletion worsened the fibrotic phenotype induced by bleomycin [154]. The specific PG downstream of COX2 and responsible for these observed effects seems to be cell–type specific. Hematopoietic cells express PGD, and PGD synthase knockout mice had higher degree of collagen deposition and increased mRNA expression of TNFα and other pro–inflammatory mediators [155]. Fibroblasts from IPF patients synthesize lower amounts of PGE2 than control subjects, and they are also less responsive to treatments with PGE2 [156]. The mechanisms for the antifibrotic effects of PGE2 are unclear, with different studies having shown even contradictory conclusions in some cases. Mice knockout for PGE synthase 1 (PGES1) were unable to increase PGE2 amounts after bleomycin. However, no differences were observed in inflammation, fibrosis and pulmonary loss of function between WT and PGES1^−/−^ mice. Similarly, knocking out PGE2 or its receptors EP2 or EP4 did not alter the course of bleomycin–induced fibrosis. In contrast, knocking out the receptor for PGI2 phenocopied COX2^−/−^ mice, suggesting that the antifibrotic effects downstream COX2 are attributable to PGI2 and not PGE2. However, other experiments have shown that the responsiveness to PGE2 differs according to fibrosis etiology, and that there is significant inter–patient variability [157]. This could be related to the ability of the cells to successfully trigger local signal events through PGE2 and its receptor EP2. Fibroblasts from human and murine fibrotic lungs showed lower expression of EP2 and this was associated to its promoter’s hypermethylation [158]. In vivo, administration of PGE2 prior to bleomycin–induced fibrosis offered protective effects against decreased pulmonary function and increased collagen production. However, there was no therapeutic effect, as experimental fibrosis developed equally in mice treated with saline or PGE2 after fibrosis had been induced with bleomycin [159, 160]. TGFβ stimulation of a human fibroblast cell line altered the expression of over 1000 genes, and treatment with PGE2 reversed multiple of these changes, especially those involved in the development of a myofibroblast phenotype [161, 162]. These effects were reproducible in a cell line of fetal fibroblasts and seemed to be mediated by receptor EP2.

A different mechanism for the antifibrotic effects of PGE2 involves the activation of plasminogen and plasminogen activator system [163]. PAI–1^−/−^ mice showed increased production of PGE2 in the lung. Treating primary fibroblasts from control and bleomycin–treated mice with both uPA and plasminogen together, but not when separated, increased PGE2 secretion and COX2 expression. The authors suggested an axis plasminogen/plasmin/extracellular hepatocyte growth factor (HGF)/HGF receptor as antifibrotic mechanism. But other mechanisms independent of HGF have also been proposed [164]. Plasminogen activation and plasmin enhanced protein kinase A (PKA) signaling by decreasing protein phosphatase 2A (PP2A) activity, thus leading to sustained phosphorylated status of PKA substrates. Suppression of PP2A activity in IPF–patient derived cell lines of fibroblasts helped overcome the resistance to PGE2 treatment. Finally, it was concluded that PAI–1^−/−^ mice are resistant to experimental fibrosis because of a sustained activation of plasminogen and enhanced proteolytic activity of uPA and downstream activation of plasmin.

Leukotrienes are lipid metabolites also derived from arachidonic acid and with signaling properties in pulmonary fibrosis. Leukotriene–deficient 5–lipooxigenase knockout mice (5–LO^−/−^ mice) were resistant to FITC–triggered experimental fibrosis potentially due to their inability to trigger receptor–mediated proliferation of basal fibrocytes [165].

Lipoxins, resolvins, protectins and maresins are other eicosanoid lipids with different chemical structures grouped under the umbrella term “resolving mediators” due to their roles in the resolution of inflammation. Resolvins and maresins derive from docosahexaenoic acid (DHA). In mice, resolvins ameliorated the bleomycin–induced increases in BAL cellularity and profibrotic cytokines, they improved Aschroft fibrosis score and also restored the levels of MMP9 to pre–bleomycin levels [166]. Maresin 1, which is produced by activated macrophages during inflammation, suppressed EMT by suppressing Smad2/3 and Akt signaling in vivo [167]. In vitro, Maresin 1 prevented TGFβ1–induced fibroblast proliferation, migration and differentiation into myofibroblast [168].

Amongst sphingolipids, Sphingosine–1–phosphate (S1P) is perhaps the best studied lipid in pulmonary fibrosis. Sphingolipids are essential constituents of plasma membranes and regulate important cellular functions, including apoptosis and proliferation. The balance of intracellular sphingomyelin and ceramide is crucial in inflammatory conditions, and the roles of ceramides and sphingolipids in chronic lung disease have been reviewed recently [169, 170]. S1P is synthesized by phosphorylation of sphingosine by sphingosine kinase 1 (SPK–1) and Sphingosine Kinase 2 (SPK–2). S1P can be secreted as a potent water–soluble signaling lipid capable of activating G–proteins coupled receptors in the target cells. Signaling can be stopped by degradation of S1P through the action of S1P phosphatases and S1P lyase [171].

S1P and SPK–1 were higher in BAL of IPF patients, and the expression of SPK1 inversely correlated with pulmonary function measures such as diffusing capacity for carbon monoxide (DLCO), forced expiratory volume in 1 s (FEV1), and FVC. Bleomycin–induced fibrosis in animal models showed consistent phenotypes, and genetic deletion of SPK1 improved pulmonary fibrosis, while deletion of S1PL worsened it [172, 173]. In vitro studies to seek the mechanism showed that fibroblast treatment with TGFβ increased S1P as well as expression of S1PL through SMAD3 activation. Overexpression of S1PL restored intracellular S1P levels through modulation of autophagy. Together, these data highlight the relevance of fine regulation of S1P signaling during disease (Table 1) [174].

## Role of alveolar lipids during pulmonary infections

The lungs are constantly exposed to microbes that enter the respiratory tract by aspiration. An effective pulmonary host defense is able to tolerate a low level of microbial invasion. However, the development of respiratory infections may occur in the event of defect in host defense, an overwhelming inoculum, or exposure to a virulent microorganism. Respiratory infections can be broadly categorized into upper respiratory tract infections, affecting the mouth, nose, sinuses, throat, larynx and trachea, and lower respiratory tract infections, affecting the lower airways, bronchi and alveoli. Upper respiratory tract infections typically present as common colds, influenzas, epiglottitis, sinusitis, and pharyngitis. Lower respiratory tract infections typically include bronchitis, bronchiolitis and pneumonia. Some of the deadliest infections, including influenza, pneumonia, and tuberculosis, exploit properties of lipids to enhance their propagation and pathogenicity, making lipid metabolism a critical player in the pathogenesis of pulmonary infections.

Pneumonia encompasses an umbrella of conditions that may arise from many etiologies, including bacterial, viral, mycoplasmal, fungal, lipoid, and aspiration of other exogenous substances. The most common form of pneumonia is bacterial pneumonia, and it alters host lipid composition and transport. BAL from human subjects with bacterial pneumonia showed mild decrease in total surfactant PL, marked decrease in glycerophospholipid and increase in phosphatidylinositol and sphingomyelin amounts (Table 1) [67]. While much remains to be uncovered regarding the consequences of surfactant alterations, these changes in the surfactant lipidome were thought to interfere with the surface tension reduction and antimicrobial functions [67, 175]. In addition to surfactant lipids, other BAL lipids also changed during pneumonia. Cardiolipin, a mitochondrial–specific lipid, was significantly elevated in BAL from infected humans and mice, and this markedly increased alveolar surface tension, decreasing lung compliance and increased IL–10 and BAL protein concentration [176]. Enrichment of cardiolipin in BAL was also correlated with decreased BAL surfactant proteins SP–A and SP–C and disruption of alveolar architecture. One proposed mechanism was that cardiolipin interferes with the packaging of surfactant DPPC, thereby increasing surface tension. The amount of cardiolipin in the alveolar lumen is regulated by the cardiolipin transporter ATP8b1, which internalizes and sequesters cardiolipin from the extracellular space. Mice bearing a missense mutant form of ATP8b1 present in many humans showed increased susceptibility to infection and infection–induced lung injury [176]. These increases in BAL cardiolipin content are also consistent with other types of acute lung injury [177, 178].

Interestingly, not only does bacterial pneumonia influence host surfactant composition, but exposure of bacteria to specific surfactant lipids was found to alter bacterial transcriptomics suggesting novel mechanisms of host–pathogen interaction. *K. pneumoniae* MGH78578 exposed to purified PC and cholesterol in vitro showed increased transcriptional levels of genes involved in capsule synthesis, lipopolysaccharide modification, antibiotic resistance, biofilm formation, and metabolism [179]. This increase in virulence gene expression may be especially relevant in cases of PAP and associated surfactant lipid accumulation. Mechanistic studies are needed in this field to determine the potential roles of the different surfactant lipids in different types of infection.

Lipopolysaccharide (LPS) is a major immunogenic constituent of the Gram–negative bacterial cell membrane. Accordingly, LPS is recognized by the host immune system, including TLRs, and triggers the cellular release of pro–inflammatory cytokines, eicosanoids, and potent vasodilators. Structurally, LPS is composed of Lipid A and two different oligosaccharides. Lipid A contains multiple fatty acid chains, and can interact with other hydrophobic lipids. Indeed, LPS interacts with pulmonary surfactant and inactivates it. LPS–surfactant complexing was shown in pneumonia and was proposed to contribute to its pathophysiology [180, 181]. Re–LPS, the minimal form of LPS required for bacterial growth in vitro, interacted with DPPC and caused DPPC monolayers to disperse and fluidize, altering their surface tension reducing properties [182]. Whole LPS also exerted this fluidizing effect on films in vitro, preventing lipid packing when they were compressed [183] and also prevented cholesterol packing in vitro [184].

LPS can also interact with SP–A and SP–D. In fact, SP–A specifically recognizes LPS lipid A [185, 186]. SP–A and SP–D play an important role in the innate immune response to pathogen–associated molecular patterns (PAMPs), and they can modulate the host response to LPS challenge by altering host cytokine release [187–189] and by scavenging LPS, minimizing LPS–mediated surfactant clumping [183]. In addition, SP–A and SP–D also destabilized the bacterial cell membrane [190].

Lipoid pneumonia is a rare condition characterized by the accumulation of endogenous or exogenous lipids in the alveoli and has been described as a precursor for other respiratory conditions, including PAP [191]. Lipoid pneumonia often presents with sudden onset of nonspecific respiratory symptoms and may be diagnostically confirmed by the demonstration of lipid–engorged macrophages in BAL, sputum, or lung tissue. Treatment for this rare condition is ill–defined, but whole lung lavage and corticosteroid administration have been described as potential treatments for advanced or recurrent lipoid pneumonia [192]. Endogenous lipoid pneumonia may occur following damage to alveolar cells that causes release of lipids into the alveolar lumen [193]. The influx of cellular lipids into the airspace and compensatory uptake by AM activates an immune response that often leads to the progression of pulmonary disease. Exogenous lipoid pneumonia, on the other hand, has traditionally been linked to the aspiration of oily substances; however, this condition is now being linked to e–cigarette vaping, which has an increasing incidence [194, 195]. In a case study, vaping–associate lipoid pneumonia was associated to lung accumulation of vegetable glycerin, a major e–cigarette liquid component, causing dysregulation of AM lipid metabolism [196]. Moreover, the dysregulation of endogenous and exogenous alveolar lipid uptake by macrophages disrupts surfactant clearing and induces an immune response.

A special example of alterations in pulmonary lipid metabolism by an external hydrophobic agent is Amiodarone, a highly effective anti–arrhythmic drug that has potential serious side effects and toxicities, with pulmonary toxicity incidence being around 7% [197]. Apoptosis of alveolar T2C is a major contributor in amiodarone induced lung injury. In a murine model of amiodarone treatment, electron microscopy showed T2C hyperplasia and extensive lung fibrosis. Surfactant phospholipids and proteins accumulated intracellularly over time [134]. Using polarized light microscopy, Haller et al. showed that amiodarone induced alterations in lamellar bodies, leading to impaired pulmonary surfactant packing and function [198]. High resolution subcellular imaging also showed amiodarone accumulation in lysosomes of lung macrophages [199]. Other mechanisms are immune–related and activation of the renin angiotensin system [200].

Viral infections can alter a myriad of metabolic pathways in the host. In a recent untargeted metabolomic study of serum from a cohort of adult subjects infected with the influenza virus, 26 different host metabolites showed differential alterations upon infection. The metabolic pathways affected included FA biosynthesis and oxidation, PL metabolism, steroid hormone metabolism, and nucleotide and amino acid synthesis (Table 1). These data point at the effects a pulmonary infection can exert in circulating lipids [201].

Influenza infections course with inflammation and NSAIDs are a common first line of treatment. The efficacy of additional anti–inflammatory candidates is currently being tested for the treatment of influenza. PGE2 and its synthase PGES–1 are attractive targets for the development of new drugs. In mice, one of the evaluated compounds successfully decreased the expression of cytokines and other pro–inflammatory genes and provided improvement in infection resolution [202]. Protectin D1 (PD1) is a DHA–derived pro–resolving mediator synthesized by the 12/15–LOX pathway that potently inhibits viral replication by inhibition of RNA export from the nucleus of infected cells. In mice, deletion of 12/15–LOX increased viral replication and disease propagation [203]. Indeed, 12/15–LOX and its metabolites were protective during inflammation resolution after influenza infection [204].

The virulence of the influenza virus can be modulated by interactions between the host lipids and the viral proteins. After initial infection and proliferation within the host cell, influenza viral particle assembly is a critical step. Viral particle assembly is orchestrated through the viral matrix protein M1, which must contact plasma membrane lipids for effective viral packaging and release. The lipid composition of the inner and outer leaflets of the plasma membrane was essential for influenza virulence. Specifically, M1 bound phosphatidylserine with high affinity and facilitated viral assembly [205]. The influenza protein M2 also takes advantage of cholesterol in the plasma membrane, which can bind the amphipathic helices of M2 to stabilize the protein and induce a conformational change. This conformational change confers an increased ability of M2 to induce the membrane curvature required for viral budding [206]. Thus, influenza increases its virulence through manipulation of host alveolar lipids.

Influenza not only coopts host lipids to its advantage, it also utilizes its own lipid packaging to enhance its virulence. Efforts to profile the lipid composition of influenza envelope have found a high degree of structural flexibility; this flexibility, conferred by the pathogen’s lipid profile was found to substantially protect the virus, and increase puncturing capacity of target cells [207].

Tuberculosis (TB), a leading cause of death worldwide, causes an estimated 1.2 million deaths and 10 million incident cases in 2018 according to the World Health Organization (WHO) [208]. TB transmission occurs through the inhalation of *Mycobacterium tuberculosis* (*Mtb*)–containing aerosolized liquid droplets by the new host [209]. One third of the world’s population is estimated to harbor the latent *Mtb* pathogen, but reactivation of the pathogen and development of active TB occurs only in approximately 5–10% of these individuals with latent TB, frequently as a result of immunodepression.

The lipids present in the host environment are important factors contributing to *Mtb* pathogenesis. *Mtb* H37Rv cultured in lipid–rich media showed increased expression of 368 genes, many of which are involved in conferring drug resistance and increasing the pathogen’s longevity [210]. In fact, *Mtb* preferentially metabolizes host–derived lipids, namely triglycerides and cholesterol, in order to perform at optimum virulence capacity. Defects in the bacterial catabolism pathways of these fuels constrains *Mtb* development [211]. In addition, eicosanoids also modulate host responses to *Mtb* infection. Infected PGE2 receptor–deficient mice accumulate higher pathogen loads than WT mice, suggesting that the host’s PGE2 is protective against *Mtb* infection [212, 213]. In contrast, Lipoxin A4 (LXA4) and other 5–lipoxygenase products enhance *Mtb* propagation in the host [213, 214].

Interestingly, over 250 genes in the *Mtb* genome encode proteins that participate in lipid metabolism, representing a vast proportion of the pathogen’s genome, and many of the lipids in these metabolic pathways are part of the bacterial cell wall [215]. A study profiling the evolution of *Mtb* strains comparing modern and ancestral bacteria, showed that modern *Mtb* contains more apolar cell–surface lipids, with decreased proportions of exposed polar lipids [216]. The effect of these changes is an enhanced capacity of the pathogen for aerosolized transmission. The cell wall lipid phthiocerol dimycocerosates (PDIM) was one of the first virulence factors to be identified in *M. tuberculosis*, and it is ubiquitously expressed in patient isolates. PDIM–deficient H37Rv mutants demonstrated significantly attenuated virulence in guinea pigs [217]. Two *Mtb* cell wall lipids, diacylated sulfoglycolipids and the phosphatidyl–myo–inositol dimannosides are currently being used as antigens in preclinical trials for a vaccine development for TB [218]. Altogether, these findings illustrate the important roles that the host and viral lipids play in the development of respiratory infection with various pathogens.

While this paper was under revision, an outbreak of a new infectious respiratory illness, named COVID–19, was declared Public Health emergency as it reached pandemic levels. The knowledge on the biology of COVID–19 is extremely limited at this moment [219]. The transmission seems to occur through droplets and symptoms generally included a high fever, headache, cough, fatigue, and respiratory distress that can quickly evolve to pneumonia and ARDS. The virus responsible for this new disease was named SARS–CoV–2, for its genetic similarity to SARS–CoV, the cause of the severe acute respiratory syndrome (SARS) outbreak first reported in February of 2003. Studies of human lung tissues from individuals with COVID–19 and SARS reported similar cell tropism for the two viruses: alveolar T1C, T2C and AM [220, 221]. Biopsied SARS and COVID–19–infected lungs document diffuse alveolar damage as a major hallmark of the diseases, as well as gross organizational changes in the alveoli and interstitial fibrosis by immunofluorescence [222, 223]. SARS infects T2C and eventually induces their apoptosis, spreading to adjacent alveoli, and it has been proposed that SARS–CoV–2 follows a similar path [224]. COVID–19–infected lung tissue shows T2C hyperplasia and erosion of the T1C epithelial lining [222], and autopsied lungs also confirm these findings, with clear T2C proliferation and alveolar barrier break down [221]. At the moment, there is no knowledge on the long term–effects of SARS–CoV–2 infection on the T2C functions of surfactant homeostasis and of alveolar repair.

## Lipids during acute lung injury and acute respiratory distress syndrome

Animal–derived surfactant therapy was first used clinically in 1980 to treat 10 preterm infants with IRDS [66]. Since then, a number of studies and randomized control trials have deemed the use of animal–derived surfactants, most commonly bovine– or porcine–derived, successful [65, 225–236]. The positive therapeutic effect of these surfactants in the treatment of IRDS is unambiguous, but concerns about the potential infectivity and antigenicity of animal–derived surfactant, as well as production and cost have encouraged the development of synthetic options, which could potentially expand the therapeutic applications to adult patients with ARDS.

Therapeutic surfactant formulations have evolved over the past few decades to yield improved therapeutic benefit to neonate patients. Early clinical trials using nebulized synthetic DPPC for the treatment of IRDS showed negative results [237, 238], and these were attributed to limited delivery of DPPC to the alveoli. Nearly 20 years later, a multicenter trial successfully piloted the use of pumactant, a synthetic surfactant preparation composed of a combination of DPPC and phosphatidylglycerol [239]. Since then, additional synthetic surfactant preparations have been formulated. Colfosceril palmitate was an FDA–approved protein–free surfactant that showed positive results in randomized control trials [240]. However, a meta–analysis comparing synthetic and animal–derived surfactants reported an inferiority of synthetic surfactants owing to their lack of SP–B and SP–C, and for this reason colfosceril palmitate is no longer used [241].

Second–generation synthetic surfactants incorporated molecules that mimic surfactant protein function. A preparation containing recombinant SP–C showed moderate symptomatic improvement in adults with ALI, but did not improve survival [242]. Other synthetic preparations contain different compounds designed to mimic SP–B activity, and have improved stability and resistance to inactivation [243]. Lucinactant was FDA– approved in 2012 and contains an SP–B–like peptide. The efficacy of these second–generation, protein–containing synthetic surfactants were shown in randomized clinical trials to be comparable to that of animal–derived surfactants for the treatment of IRDS [244, 245]. Additional synthetic surfactant preparations are currently being investigated and optimized for resisting the surfactant inhibitory conditions in the alveolar microenviroment of the patient with IRDS [246].

Indeed, decreased pulmonary compliance and increased edema are major pathophysiological findings also in ARDS, and decreased surfactant PL as well as incorporation of PL into polymerized fibrin contribute to this pathophysiology (Table 1) [247]. BAL phosphatidylglycerol was also decreased in ARDS with increased BAL surface tension [69]. Experimental supplementation with phosphatidylglycerol in a neonatal piglet ARDS model reduced IL–6 and alveolar apoptosis, and preserved the alveolar–capillary barrier, thus decreasing pulmonary edema [248].

The role of sphingolipids in acute lung injury (ALI) and ARDS is still unclear and controversial. S1P has been shown to have protective effects against ALI, but the downstream effects of SPK1 and SPK2 in inflammation and lung injury seem to be related to the type of initial insult. SPK1^−/−^ mice were more susceptible to lipopolysaccharide (LPS)–induced lung injury than WT mice, showing increased neutrophil infiltration and endothelial leakage, as well as increased inflammatory cell numbers in BAL [249]. However, *Escherichia coli* lung infection resulted in enhanced progression of disease in SPK2 but not SPK1^−/−^ mice, independently of neutrophil recruitment and effector functions [250]. In mice with S1PL inhibition or genetic deletion and subsequent increase in S1P levels, LPS challenge had low efficiency at eliciting lung injury and inflammation. Moreover, down–regulation of S1PL expression in human lung endothelial cells decreased LPS–induced endothelial barrier disruption and IL–6 production, suggesting that S1PL might be a potential therapeutic target in ALI and ARDS [251]. Further studies are required to evaluate these hypotheses.

The efficacy of aerosolized surfactant therapy has been piloted in clinical trials for ARDS. In a randomized prospective control trial piloting the use of exogenous surfactant therapy to treat chronic bronchitis, aerosolized surfactant therapy was found to improve subject pulmonary function and improve sputum transport by respiratory cilia [90]. The potential of surfactant for the treatment of ARDS is still unclear.

## Summary and conclusions

**Fig. 2:**
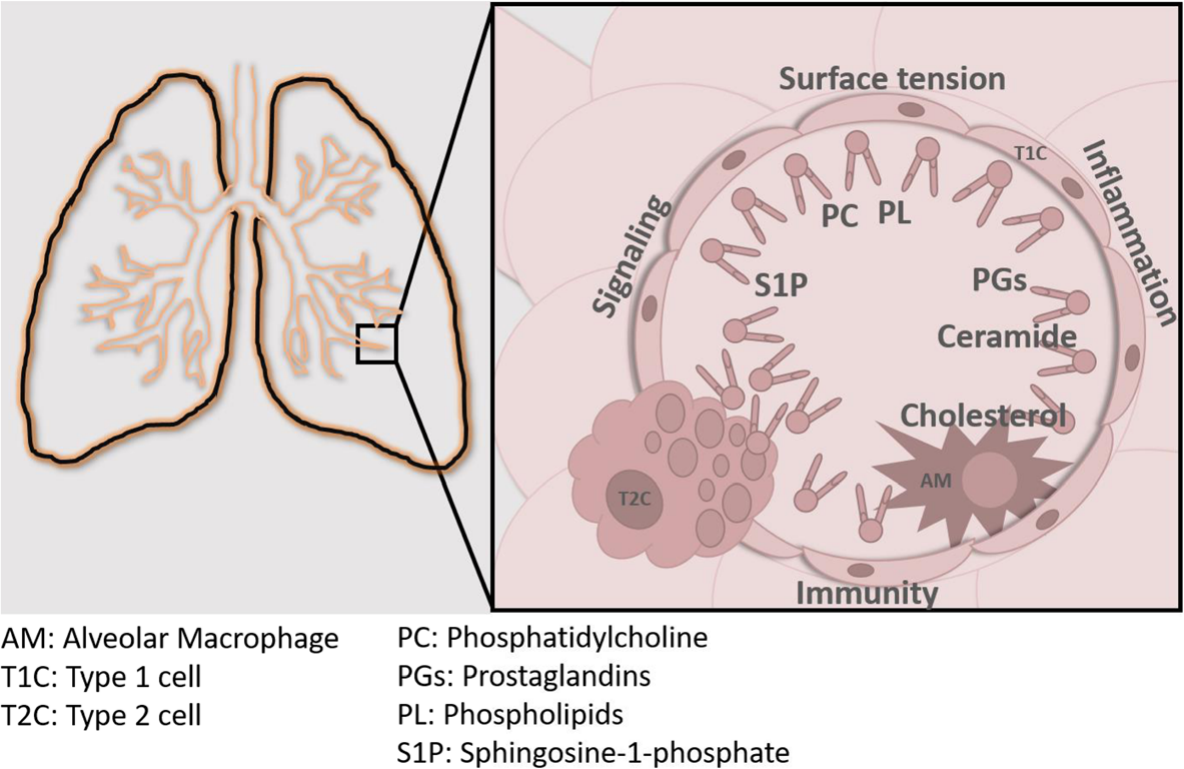
Alveolar lipids in pulmonary homeostasis. Schematic representation of alveolar cell types and the main lipids that partake in multiple functions during pulmonary homeostasis and pathophysiological conditions.

The lung parenchyma consists of multiple cell types with specific structures and functions, conferring a remarkable complexity in the study of the pathophysiology of pulmonary disease (Fig. 2).

Alveolar surfactant is essential for respiratory function and it is mostly composed of phospholipid, with minor but very specific amounts of other lipids and surfactant proteins. Qualitative and quantitative changes in the surfactant lipids are involved in multiple adult pulmonary pathologies (Table 1). COPD, exposure to pollution and smoke, and the use of cigarettes result in detrimental changes of the surfactant lipids, the surfactant–synthesizing T2C and the surfactant–clearing alveolar macrophages. IPF can arise as a result of prolonged damage to the T2C, and the cell–specific transcriptomic signature of this disease shows profound alterations in intracellular lipid metabolism in T2C and in macrophages. Not only are surfactant lipid metabolic pathways impacted, but bioactive sphingolipids and prostaglandins also show mechanistic involvement in IPF models. Alveolar lipids also partake in the pathophysiology of infectious diseases and ARDS.

The current limitations on the study of pulmonary lipids include the vast complexity that lipid metabolism can quickly acquire. Mass spectrometry now allows sensitive detection of specific lipid species, allowing for more detailed analysis, and the new challenge is the interpretation of such lipidomic data, taking into account that lipids are often metabolites, and as such, they may not accumulate and instead be in a state of flux. Another important limitation is the cellular heterogeneity of the lung. Different cell types may regulate lipid metabolism differently according to their specific functions, despite cells interacting with each other and residing in the same niche. Specific modulation of metabolism in specific cell populations is a challenge yet unresolved. For analytical goals, techniques like single cell sequencing allow transcriptomic assessment of different cell populations, but for lipid biology the techniques are restricted to whole tissue assessment, or cell–separations by combinations of differential centrifugation and surface marker–based selection.

Much work remains to be done to elucidate the details of lung lipid metabolism and signaling with the ultimate goal of developing new therapies, but this is a promising field that will likely expand in the years to come.

## Data Availability

Not applicable.

## Abbreviations

AA: Arachidonic acid
ABC: ATP–binding cassette
AM: Alveolar macrophage
ARDS: Acute respiratory distress syndrome
BMP: Bis monoacyl phosphoglycerate
BAL: Bronchoalveolar lavage
CCTα: CTP:phosphocholine cytidyltransferase alpha
COPD: Chronic obstructive pulmonary disease
COX: Cyclooxygenase
DLCO: Diffusing capacity for carbon monoxide
DGAT: Diglyceride acyltransferase
DHA: Docosahexaenoic acid
DPPC: Dipalmitoyl phosphatidylcholine
ECM: Extracellular matrix
Elovl6: Elongation of very long chain fatty acids protein 6
EMT: Epithelial–mesenchymal transition
ENDS: Electronic nicotine delivery system
FA: Fatty acid
FAS: Fatty acid synthase
FEV_1_: Forced expiratory volume in 1 s
FVC: Forced vital capacity
GPIHBP1: Glycosylphosphatidylinositol–anchored high density lipoprotein–binding protein 1
GOLGA2: Golgin A2
GM–CSF: Granulocyte macrophage colony stimulating factor
HFD: High fat diet
IL: Interleukin
IFN–γ: Interferon–γ
IPF: Idiopathic pulmonary fibrosis
IRDS: Infant respiratory distress syndrome
LFA: Lymphocyte function–associated antigen
LPCAT1: Lysophosphatidylcholine acyltransferase 1
LpL: Lipoprotein lipase
LPS: Lipopolysaccharide
LysoPC: Lysophosphatidylcholine
LOX: Lipoxygenase
LXA4: Lipoxin A4
MAPK: Mitogen–activated protein kinase
PAI–1: Plasminogen activator inhibitor
PGES1: PGE synthase 1
PC: Phosphatidylcholine
PD1: Protectin D1
PDIM: Phthiocerol dimycocerosates
PG: Prostaglandin
PKA: Protein kinase A
PL: Phospholipid
PLA_2_: Phospholipase A2
POG: Palmitoyl–oleoyl–phosphatidylglycerol
PP2A: Protein phosphatase 2A
S1P: Sphingosine–1–phosphate
SCD1: Stearoyl–CoA desaturase 1
SP: Surfactant protein
SPK: Sphingosine kinase
SRA: Scavenger receptor A
T1C: Alveolar type 1 cells
T2C: Alveolar type 2 cells
TB: Tuberculosis
TGFβ: Transforming growth factor beta
TLR: Toll–like receptor
TNF–α: Tumor necrosis factor–α
TX: Thromboxane
uPA: Urokinase–type plasminogen activator
URI: Upper respiratory infection
WT: Wild–type
